# Comorbidity Patterns of Older Lung Cancer Patients in Northeast China: An Association Rules Analysis Based on Electronic Medical Records

**DOI:** 10.3390/ijerph17239119

**Published:** 2020-12-06

**Authors:** Jia Feng, Xiao-min Mu, Ling-ling Ma, Wei Wang

**Affiliations:** Department of Medical Informatics, School of Public Health, Jilin University, 13002100 Changchun, China; muxm18@mails.jlu.edu.cn (X.-m.M.); mall19@mails.jlu.edu.cn (L.-l.M.); w_w@jlu.edu.cn (W.W.)

**Keywords:** comorbidity, association rules, lung cancer, aging, chronic disease management

## Abstract

Purposes: This study aims to identify the comorbidity patterns of older men with lung cancer in China. Methods: We analyzed the electronic medical records (EMRs) of lung cancer patients over age 65 in the Jilin Province of China. The data studied were obtained from 20 hospitals of Jilin Province in 2018. In total, 1510 patients were identified. We conducted a rank–frequency analysis and social network analysis to identify the predominant comorbidities and comorbidity networks. We applied the association rules to mine the comorbidity combination with the values of confidence and lift. A heatmap was utilized to visualize the rules. Results: Our analyses discovered that (1) there were 31 additional medical conditions in older patients with lung cancer. The most frequent comorbidities were pneumonia, cerebral infarction, and hypertension. (2) The network-based analysis revealed seven subnetworks. (3) The association rules analysis provided 41 interesting rules. The results revealed that hypertension, ischemic cardiomyopathy, and pneumonia are the most frequent comorbid combinations. Heart failure may not have a strong implicating role in these comorbidity patterns. Cerebral infarction was rarely combined with other diseases. In addition, glycoprotein metabolism disorder comorbid with hyponatremia or hypokalemia increased the risk of anemia by more than eight times in older lung cancer patients. Conclusions: This study provides evidence on the comorbidity patterns of older men with lung cancer in China. Understanding the comorbidity patterns of older patients with lung cancer can assist clinicians in their diagnoses and contribute to developing healthcare policies, as well as allocating resources.

## 1. Introduction

Over half of the cancer patients aged 65 or older have at least one chronic condition, and almost one-quarter have at least four [1], generally referred to as comorbidities, which have become an issue of growing importance due to the increasing number of older cancer patients [2,3]. As age increases, the comorbidity issues for older lung cancer patients become more prominent compared to those of younger patients. Age is a key negative prognostic factor, like tumor stage and the state of a patient’s health [4]. It has been widely observed that the comorbidities of cancer patients influence both prognosis and treatment plans [5]. While general knowledge has been established about the complexity created by the issue of comorbidities among older patients, very few studies have been published focusing on the disease distribution and comorbidity patterns [6].

The majority of the published studies on comorbidities seek to determine, based on large-scale health information databases and/or epidemiological studies, the prevalence [7,8,9], the relative factors [8,9,10,11,12], and the common patterns and clusters of comorbidities, as well as the relevant healthcare [10,13,14,15], which can provide insights into disease pathogenesis. In addition, some research focuses on the impact of comorbidities on the clinical outcomes of different diseases [16,17,18,19,20]. In terms of the comorbidity studies of older adults with lung cancer, published studies have predominantly focused on the prevalence of two co-occurring morbidities, and no comprehensive comorbid patterns have been reported to the best of our knowledge.

The rapidly growing electronic medical records and epidemiological data have made it possible to conduct comprehensive comorbidity analyses. Lung cancer is the most common cancer and the leading cause of cancer-related deaths in China, especially in Northeast China [21]. In this paper, we present a study on identifying the comorbidity pattens of older lung cancer patients in Northeast China. Numerous analytical methods have been utilized to study the comorbidity issue, such as prevalence statistics [22,23], the proportion of comorbidity pairs in the population [24,25], a correlation analysis [26,27], and a clustering analysis [28,29]. In recent years, machine learning methodology has been applied to comorbidity analyses, e.g., to predict comorbidities and clinical outcomes [30,31]. Association rule mining (ARM) can discover the relationships in large databases, as proposed by Agrawal et al. [32]. ARM is well-researched and commonly used in pattern mining, such as for determining clinical decisions [33], disease factors [34], and drug utilization [35]. The support and confidence, which are measures of the rules, are used to assess the relationships among comorbidities [36,37,38].

We adopted the rank–frequency analysis method to identify the main morbidities and conducted association rule- and network-based analyses to model the comorbidities.

## 2. Materials and Methods

### 2.1. Study Population and Data Collection

The data studied are the electronic medical records (EMRs) of Jilin Province, China in 2018. Jilin Province is located in the northeast of China. Due to the area’s weather and environmental pollution, the northeast area has the highest lung cancer incidence rate [39] in China. The clinical records of enrollees were taken from the tertiary grade A hospitals [40] in Jilin Province. The International Classification of Diseases 10th revision (ICD-10) is used in the public hospital diagnosis system of Jilin Province. All categories of comorbidities in this study followed the original categories of the ICD-10 system. All data used here were obtained from patients with lung cancer who were at least 65 years old. We removed patients with missing information on their gender, hospital, or case number and set the case number combined with gender and age as the primary key. Ethical approval to conduct this study was obtained from the Ethics Committee of the School of Public Health, Jilin University (grant number: ethical review 2020-02-01).

### 2.2. Statistical Models and Discovery of Association Rules

#### 2.2.1. Rank—frequency Analysis

Here, we focused on the predominant comorbidities among older lung cancer patients. The Pareto principle, or the 80–20 rule, was applied here, which suggests that 80% of the impact comes from 20% of the potential causes [41]. This study hypothesized that the distribution of comorbidity is consistent with this general principle. To identify the major morbidities, this study applied a rank–frequency analysis, which is the distribution of size by rank in decreasing order of size. A rank–frequency distribution can generally be segmented into ranges, each having similar values. The most common distribution splits the distribution into two as the head and tail. We considered the head to consist of the first *p*-ranks; hence, 80% of the overall population can be explained by the first 20% of the heads based on the Pareto principle. We plotted the contributing morbidities in their rank order as the absolute amounts, along with a line representing their cumulative percentage contributions to the overall population. When the line is located at 80% of the cumulative contribution, the predominant comorbidity can be identified.

#### 2.2.2. Discovery of Association Rules

Association rules are used to detect the comorbidity patterns in our study. In a clinical record database of participants, the collection of whole diagnoses and their possible combination sets are denoted as a transaction (T). The ARM method is used to extract an association rule from the T. A rule “X ⇒ Y” is derived for disease X and disease Y if X occurs in a patient and Y cooccurs in the same person [31], with X being the left-hand side (LHS) and Y being the right-hand side (RHS) of the rule.

Each association rule has three associated values: support, confidence, and lift.

- Support measures the co-occurrence frequency of X and Y in the patient dataset, i.e., the number of patients having both X and Y divided by the total number of patients, denoted as P(X, Y).
- Confidence measures the reliability of a rule—namely, the probability of seeing Y among patients with X, denoted as P(Y|X).
- Lift measures the significance of the support P(X, Y) of a rule by calculating the ratio between the observed co-occurrence frequency P(X, Y) and the expected co-occurrence frequency P(X)×P(Y) when X and Y are independent—namely, $\frac{P\left(X,Y\right)}{P\left(X\right)P\left(Y\right)}$. If the ratio is close to 1, then little information is provided by this rule. If the ratio is greater than 1, then X and Y are positively correlated; otherwise, they are negatively correlated. Overall, this method is often used to measure the interest of a rule [42].

In this study, we set the thresholds for the three parameters as follows: confidence > 0.5, lift > 2, and support > 0.01. In addition, RStudio version 3.5.1 (Lucent Technologies, Murray Hill, NJ, USA) was used to conduct and visualize the AMR analyses.

## 3. Results

### 3.1. Patient Statistics

Overall, 1510 patients were included in this study. Table 1 lists the patient statistics. The average age was 71 years, and 60.2% were male. Overall, 22.5% of the patients had no comorbidities, 15.2% had only one comorbidity, and 62.3% had two or more comorbidities.

**Figure 1:**
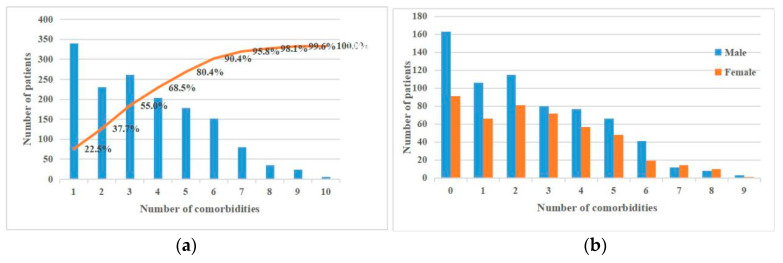
(**a**) Distribution (blue) of the number of comorbidities across all patients, where the x-axis represents the number of patients having *n* comorbidities (0≤n≤9) and the cumulative proportion distribution across all patients (orange). (**b**) Distributions of the numbers of comorbidities for men and women.

A summary of the comorbidity data across all patients is shown in Figure 1. As shown in Figure 1a, more patients had two comorbidities than one, and, as shown in Figure 1b, comorbidities were found in both males and females.

**Figure 2:**
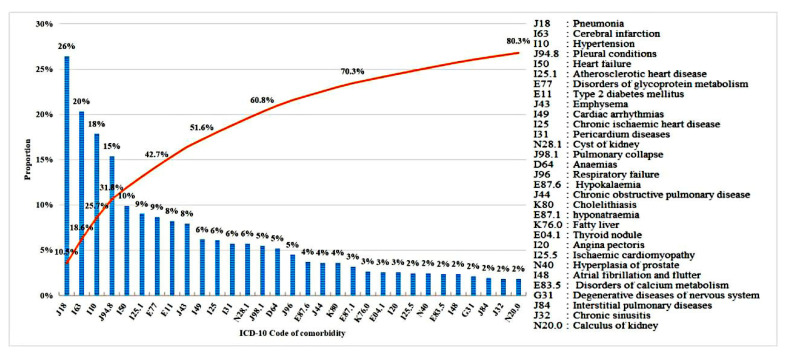
Distribution of the number of patients for each disease (**blue**) and the cumulative proportion (**orange**).

Figure 2 summarizes the number of cases for each disease observed among the patients under study and the cumulative proportion of all the participants. The most frequently observed 31 morbidities cover 80.3% of the participants, and the most frequent comorbidity is pneumonia, with cerebral infarction, hypertension, and pleural conditions being the next three most frequent comorbidities.

Table 2 shows the number of patients with lung cancer affected by a single comorbidity, as well as the respective proportions of single and multiple comorbidities. Among the most frequent comorbidities, the proportions of multiple comorbidities were found in up to 90%. Patients were likely to have other comorbidities with the following eight diseases: heart failure, chronic ischemic heart disease, hypokalemia, fatty liver, angina pectoris, disorders of calcium metabolism, and calculus of the kidney. This result may be part of the reason why more patients had two comorbidities than one.

**Figure 3:**
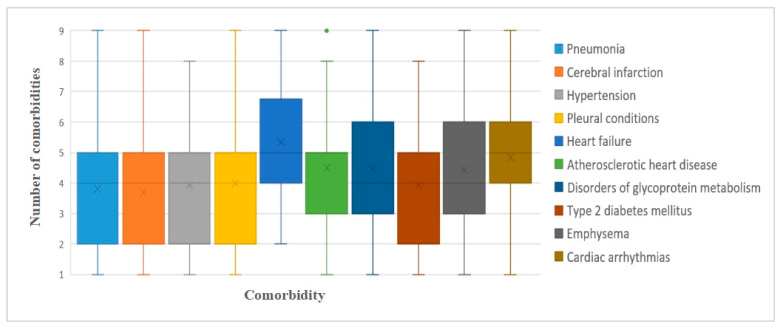
The average number of comorbidities for each co-occurring disease, ordered based on the prevalence of each disease observed in the study.

Figure 3 shows the average number of patients with the top 10 morbidities. For patients with heart failure, their number of comorbidities range from four to seven, which is higher than the average number.

### 3.2. Network-Based Analysis

**Figure 4:**
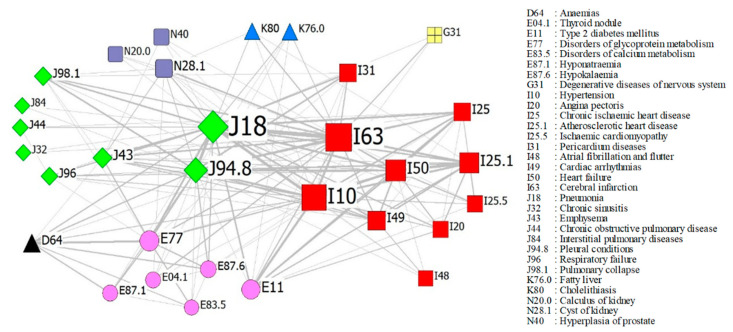
A network representation of comorbidities. Each node represents a disease, where nodes with the same shape denote a morbidity possessing the same International Classification of Diseases 10th revision (ICD-10) classification code, and an edge connects two nodes if patients were observed with this comorbidity. The size of a node is proportional to the frequency of the disease, and the width of an edge is proportional to the number of patients with the comorbidity.

We used a network representation of the comorbidities to provide a global and intuitive view of the co-occurrences among different comorbidities, as illustrated in Figure 4, showing a network of 31 comorbidities in our patient set. This figure shows the predominance of pneumonia, cerebral infarction, and hypertension among all the comorbidities. Notably, pleural conditions and heart failure were comorbid with many diseases when there were no significant differences in comorbidities (the widths of the edges are about same). This indicates that there are no specific comorbidities among lung cancer patients. A modularity analysis revealed seven subnetworks: (a) diseases of the respiratory system, including pneumonia, pleural conditions, emphysema, pulmonary collapse, respiratory failure, and chronic obstructive pulmonary disease; (b) diseases of the circulatory system—namely, cerebral infarction, hypertension, heart failure, cardiac arrhythmia, and atherosclerotic heart disease, among others; (c) endocrine and metabolic diseases—namely, type 2 diabetes, disorders of glycoprotein metabolism, hyponatremia, and hypokalemia, among others; (d) diseases of the genitourinary system, including cysts of the kidney, hyperplasia of the prostate, and calculus of the kidney; (e) diseases of the digestive system, including fatty liver and cholelithiasis; (f) anemias; and (g) degenerative diseases of the nervous system.

### 3.3. Derivation of the Association Rules

Table 3 shows the results of the 41 detected association rules. From this table, we can see there are 41 notable rules. The values of the support range from 0.01 to 0.06, which means that the rules occurred in 1% to 6% of the population that we studied. This is not a high prevalence, but the confidence and lift are quite significant, with a high value. The value of confidence ranges from 0.50 to 0.92, which means that the probability of observing Y (RHS) among patients with X (LHS) is over 50%. In addition, the values of lift range up to 8.58, and the average value is 4.91, which indicates the high significance of the rules.

**Figure 5:**
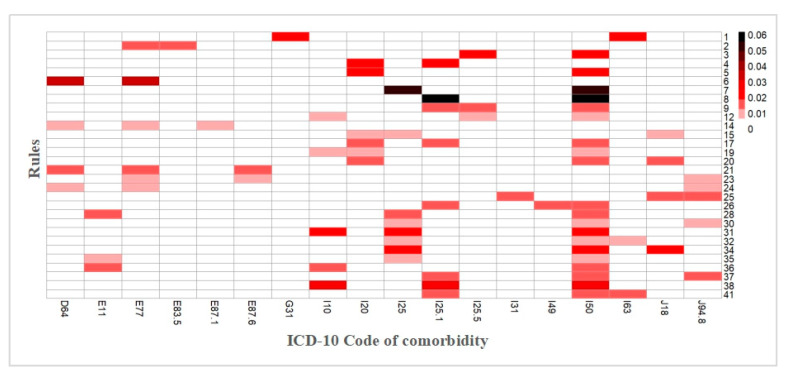
A heatmap showing the support values in the 41 derived association rules, where the rows are for the rules and the columns are for XX morbidities.

We next visualized the support values among the 41 association rules to determine which rules are the most common. Figure 5 shows the 41-rule analysis of the comorbidities, presented as a heatmap. Although each pattern is directed with an arrow, it does not mean causation between diseases, but only represents co-occurrences. To avoid confusion, We only keep one of multiple rules with the same frequent item set.For example, rules #9 and #10 used the same combination set, so we included rule #9 in the heatmap. The colors of the grid points suggest the prevalence of the morbidities indicated by the x-axis. The darker the color is, the higher the prevalence. Here, there are nine dyads and 21 triads. The most common dyads were rules #7 and #8, which include chronic ischemic heart disease (I25) and atherosclerotic heart disease (I25.1) with heart failure (I50). The most common triad was rule #38, which includes hypertension (I10) with atherosclerotic heart disease (I25.1) and heart failure (I50).

**Figure 6:**
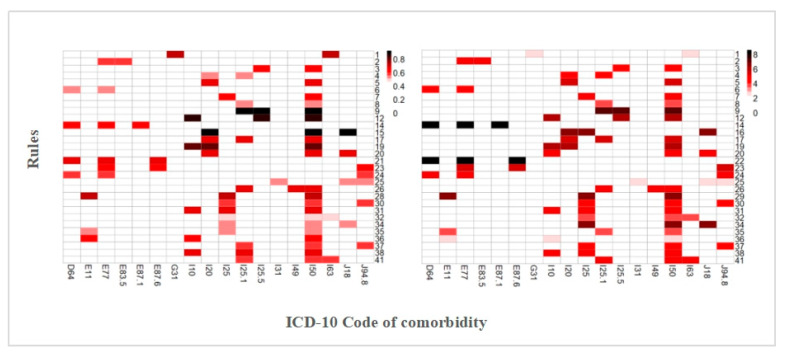
Heatmaps for the confidence and lift values observed in the 41 derived association rules. (**a**) The rows are for rules, and the columns are for XX morbidities. (**b**) Defined similar to (**a**).

Figure 6 shows the confidence and lift heatmaps of older lung cancer comorbidities. The confidence measures for lung cancer comorbidities with a value > 0.5 are shown in Figure 6a. The colors of the grid points indicate the probability of having the disease indicated by the x-axis, where the darker the color is, the higher the probability. Rule #12 indicates a higher probability of hypertension (I10), ischemic cardiomyopathy (I25.5), and pneumonia (J18). In addition, in rule #9, the combination of atherosclerotic heart disease (I25.1), ischemic cardiomyopathy (I25.5), and heart failure (I50) occurred much more frequently than expected, as did rules #15 and #19, which are heart failure (I50), angina pectoris (I20) with pneumonia (J18), and hypertension (I10). This study found that heart failure (I50) occurred in most rules. However, as a common geriatric disease, heart failure may not have a strong implicating role in the relevant comorbidity patterns. In addition, the results show that cerebral infarction (I63) is the second most frequent comorbidity (see Figure 2). However, it is rarely combined with other diseases, as shown in Figure 6.

In Figure 6b, rules #14 and #22 have the highest lifts. They occur about eight times more frequently than expected under statistical independence. Table 2 shows that disorders of glycoprotein metabolism comorbid with hyponatremia or hypokalemia increased the risk of anemia by more than eight times. Other rules with a relatively high lift, such as rules #9, #12, and #19, indicate that when hypertension is comorbid with ischemic heart diseases in a lung cancer patient, the occurrence of heart failure increases by six times. Notably, in rule #16, which applies when angina pectoris is comorbid with pneumonia, chronic ischemic heart disease occurs more frequently. In addition, type 2 diabetes mellitus (E11) and pericardium diseases (I31) were rarely combined with other diseases.

## 4. Discussion

This study conducted a comorbidity pattern analysis using a network graph and association rules to investigate the disease associations among 1510 older Chinese adults over age 65. Overall, 77.5% of the patients had comorbidities, which was significantly higher than the results for the elderly population [43,44]. Moreover, the proportions of multiple comorbidities were up to 90%, which is consistent with previous studies [21]. Further, we determined lung cancer patients to have higher proportions of multiple comorbidities and that these comorbidities are complex.

The network analysis illustration showed that there are seven subnetworks among the comorbidity networks in elderly lung cancer participants. Among diseases of the respiratory system, pneumonia is a very common disease that causes significant morbidity and mortality, especially in older patients with lung cancer [45]. There is a high prevalence of pneumonia related to cardiovascular diseases and a trend toward an increased risk of poor outcomes [46,47].

Circulatory system comorbidities are also called cardiovascular comorbidities and include cerebral infarction, hypertension, heart failure, cardiac arrhythmias, and atherosclerotic heart disease. Some studies have shown that the cardiovascular comorbidity prevalence among lung cancer patients ranges from 12.9% to 43% [48,49]. Moreover, comorbidity with cerebrovascular disorders can lead to a 20% increase of mortality in non-small cell lung cancer (NSCLC) patients, so clinicians should pay attention to this comorbidity [50]. Endocrine and metabolic comorbidities include type 2 diabetes mellitus, disorders of glycoprotein metabolism, hyponatremia, hypokalemia, etc. For lung cancer patients, those with disorders of glycoprotein metabolism or diabetes had a higher mortality than patients without this comorbidity [50]. Comorbidities associated with chronic kidney disease were also found to be a risk factor for mortality in patients with lung cancer [51,52], especially cysts of the kidney. Recently, Wenyu Wu et al. [53] conducted a retrospective cohort study and found hepatic steatosis to be an independent predictor of liver metastasis in patients with NSCLC.

Based on the analysis of AMR, the most interesting rules were related to cardiovascular comorbidities, such as angina pectoris, chronic ischemic heart disease, atherosclerotic heart disease, ischemic cardiomyopathy, and heart failure. Based on the network analysis and association rule mining, it was found that heart failure is widely comorbid with other diseases, but there is no fixed comorbidity pattern, which is consistent with previous research results [54,55]. Cerebral infarction and type 2 diabetes mellitus are very common in lung cancer patients, with an incidence of 20% and 8%, respectively; however, they rarely occur with other diseases. The incidence of cerebral infarction is also high in NSCLC, especially in patients with brain metastasis compared to those without brain metastasis [56]. In addition to the role of traditional stroke risk factors, cerebral infarctions in patients with malignant tumors have specific pathogenic mechanisms, such as ischemic stroke caused by aneurysm embolization and direct compression of the arteries in the head and neck [57].

In addition, disorders of glycoprotein metabolism comorbid with hyponatremia or hypokalemia increased the risk of anemia by more than eight times. Anemia was associated with confusion [58] and fatigue [59] in older lung cancer patients. The most common type of anemia in chronic lung disease is anemia from chronic disease [60]. Some studies suggest that disorders of glycoprotein metabolism may be useful as prognostic indicators in patients with lung cancer, such Glycoprotein nonmetastatic melanoma B (GPNMB) [61] and P-glycoprotein [62].

There are some limitations to our study. To identify the comorbidity network and patterns, this study used electronic medical records from Jilin Province in 2018, and diagnoses recorded in specific hospitals were included. Since the survival data of patients over time is not included, the effects of comorbidities on lung cancer prognosis cannot be explored.

We conducted a network analysis to mine the comorbidity subnetworks. This study only discussed the relevant contents qualitatively. However, some quantitative metrics could provide more information about the comorbidities’ patterns, such as their centrality, density, clustering coefficients, and propinquity. We will explore these quantitative metrics in future research. In the follow-up work, we will collect clinical cohort data to explore the role of clinicopathological factors, such as TNM staging and smoking history, and reveal how these factors affect lung cancer prognosis.

The association rule analysis was designed to identify combinations of morbidities. However, these combinations occurred more frequently than expected (without addressing the statistical significance). Moreover, the threshold set of association rules is experiential, and the results may vary depending on the threshold value chosen.

Notwithstanding these limitations, our study shows that the network analysis and association rules analysis can provide additional dimensions to better understand public health. Moreover, our study analyzed a population in the northeast area of China in terms of the different comorbidity patterns that should be considered when dealing with comorbidities.

## 5. Conclusions

In conclusion, our findings suggest that pneumonia, cerebral infarction, and hypertension are the most frequent comorbidities in older patients with lung cancer and that cardiovascular comorbidities are the most common comorbid combinations. Cerebral infarction and type 2 diabetes mellitus rarely occurred with other diseases. Disorders of the glycoprotein metabolism comorbid with hyponatremia or hypokalemia increased the risk of anemia in older patients with lung cancer. Understanding the comorbidity patterns of older patients with lung cancer will assist clinicians in their diagnoses. When clinicians choose an appropriate anticancer therapy, it is also necessary to assess the comorbidity of the patient. In addition, in terms of chronic disease management, comorbidity information can contribute to developing healthcare policies and allocating resources.

## Figures and Tables

**Table 1:** Patient statistics.

Characteristics	*N*	Percentage (%)
Total	1510	100
**Age(years)**		
65–74	1124	74.4
75–84	329	21.8
85 +	57	3.8
**Gender**		
Male	909	60.2
Female	601	39.8
**Nationality**		
Han	1416	93.8
Korean	68	4.5
Other	25	1.7
**Occupation**		
Farmers	246	16.3
Retired	337	22.3
Unemployed	84	5.6
Workers	55	3.6
Staff	23	1.5
Other	33	2.2
Unspecified	732	48.5
**Marital status**		
Unmarried	9	0.6
Married	1375	91.0
Death of a spouse	59	3.8
Divorce	11	0.7
Other	56	3.7
**No. of comorbidities**		
0	339	22.5
1	230	15.2
2	262	17.3
3	203	13.5
>3	496	31.5

**Table 2:** Patients affected by each disease independent of comorbidities (all cases) and by each disease with a single comorbidity and multi-comorbidities.

Disease	All Cases (*n*)	Single Comorbidity (%)	Multiple Comorbidities (%)
Pneumonia	299	11.7	88.3
Cerebral infarction	230	10.9	89.1
Hypertension	202	8.4	91.6
Pleural conditions	174	11.5	88.5
Heart failure	112	0.0	100.0
Atherosclerotic heart disease	102	3.9	96.1
Disorders of glycoprotein metabolism	98	1.0	99.0
Type 2 diabetes mellitus	93	10.8	89.2
Emphysema	90	4.4	95.6
Cardiac arrhythmias	70	1.4	98.6
Chronic ischemic heart disease	69	0.0	100.0
Pericardium diseases	65	10.8	89.2
Cyst of kidney	65	1.5	98.5
Pulmonary collapse	62	4.8	95.2
Anemia	59	3.4	96.6
Respiratory failure	51	7.8	92.2
Hypokalemia	42	0.0	100.0
Chronic obstructive pulmonary disease	41	7.3	92.7
Cholelithiasis	41	2.4	97.6
Hyponatremia	36	5.6	94.4
Fatty liver	30	0.0	100.0
Thyroid nodule	29	3.4	96.6
Angina pectoris	29	0.0	100.0
Ischemic cardiomyopathy	28	3.6	96.4
Hyperplasia of the prostate	28	3.6	96.4
Disorders of calcium metabolism	27	0.0	100.0
Atrial fibrillation and flutter	27	3.7	96.3
Degenerative diseases of the nervous system	24	0.0	100.0
Interstitial pulmonary diseases	22	13.6	86.4
Chronic sinusitis	21	4.8	95.2
Calculus of the kidney	21	0.0	100.0

**Table 3:** The association rules detected among lung cancer patients.

No.	Rules			Sup	Con	Lift
1	(Degenerative diseases of the nervous system)	=>	(Cerebral infarction)	0.02	0.71	2.60
2	(Disorders of calcium metabolism)	=>	(Disorders of glycoprotein metabolism)	0.02	0.56	4.78
3	(Ischemic cardiomyopathy)	=>	(Heart failure)	0.02	0.61	4.58
4	(Angina pectoris)	=>	(Atherosclerotic heart disease)	0.02	0.55	4.57
5	(Angina pectoris)	=>	(Heart failure)	0.02	0.69	5.20
6	(Anemias)	=>	(Disorders of glycoprotein metabolism)	0.04	0.53	4.53
7	(Chronic ischemic heart disease)	=>	(Heart failure)	0.05	0.62	4.70
8	(Atherosclerotic heart disease)	=>	(Heart failure)	0.06 *	0.51	3.84
9	(Atherosclerotic heart disease, Ischemic cardiomyopathy)	=>	(Heart failure)	0.01	0.92	6.96
10	(Ischemic cardiomyopathy, Heart failure)	=>	(Atherosclerotic heart disease)	0.01	0.71	5.84
11	(Ischemic cardiomyopathy, Heart failure)	=>	(Hypertension)	0.01	0.59	2.46
12	(Hypertension, Ischemic cardiomyopathy)	=>	(Heart failure)	0.01	0.83	6.28
13	(Anemias, Hyponatremia)	=>	(Disorders of glycoprotein metabolism)	0.01	0.60	5.17
14	(Disorders of glycoprotein metabolism, Hyponatremia)	=>	(Anemias)	0.01	0.60	8.58
15	(Angina pectoris, Chronic ischemic heart disease)	=>	(Pneumonia)	0.01	0.90	2.54
16	(Angina pectoris, Pneumonia)	=>	(Chronic ischemic heart disease)	0.01	0.53	6.48
17	(Angina pectoris, Atherosclerotic heart disease)	=>	(Heart failure)	0.01	0.69	5.18
18	(Angina pectoris, Heart failure)	=>	(Atherosclerotic heart disease)	0.01	0.55	4.55
19	(Hypertension, Angina pectoris)	=>	(Heart failure)	0.01	0.82	6.17
20	(Angina pectoris, Pneumonia)	=>	(Heart failure)	0.01	0.65	4.88
21	(Anemias, Hypokalemia)	=>	(Disorders of glycoprotein metabolism)	0.01	0.69	5.92
22	(Disorders of glycoprotein metabolism, Hypokalemia)	=>	(Anemias)	0.01	0.58	8.28
23	(Hypokalemia, Pleural conditions)	=>	(Disorders of glycoprotein metabolism)	0.01	0.64	5.54
24	(Anemias, Pleural conditions)	=>	(Disorders of glycoprotein metabolism)	0.01	0.56	4.84
25	(Pericardium diseases, Pneumonia)	=>	(Pleural conditions)	0.02	0.52	2.51
26	(Atherosclerotic heart disease, Cardiac arrhythmias)	=>	(Heart failure)	0.02	0.67	5.02
27	(Cardiac arrhythmias, Heart failure)	=>	(Atherosclerotic heart disease)	0.02	0.58	4.83
28	(Type 2 diabetes mellitus, Chronic ischemic heart disease)	=>	(Heart failure)	0.01	0.73	5.53
29	(Type 2 diabetes mellitus, Heart failure)	=>	(Chronic ischemic heart disease)	0.01	0.52	6.41
30	(Chronic ischemic heart disease, Pleural conditions)	=>	(Heart failure)	0.01	0.59	4.43
31	(Hypertension, Chronic ischemic heart disease)	=>	(Heart failure)	0.02	0.67	5.02
32	(Chronic ischemic heart disease, Cerebral infarction)	=>	(Heart failure)	0.01	0.50	3.77
33	(Chronic ischemic heart disease, Pneumonia)	=>	(Heart failure)	0.02	0.53	4.00
34	(Heart failure, Pneumonia)	=>	(Chronic ischemic heart disease)	0.02	0.55	6.71
35	(Type 2 diabetes mellitus, Atherosclerotic heart disease)	=>	(Heart failure)	0.01	0.53	3.99
36	(Type 2 diabetes mellitus, Heart failure)	=>	(Hypertension)	0.02	0.62	2.59
37	(Atherosclerotic heart disease, Pleural conditions)	=>	(Heart failure)	0.02	0.59	4.45
38	(Hypertension, Atherosclerotic heart disease)	=>	(Heart failure)	0.03	0.67	5.02
39	(Hypertension, Heart failure)	=>	(Atherosclerotic heart disease)	0.03	0.50	4.14
40	(Atherosclerotic heart disease, Cerebral infarction)	=>	(Heart failure)	0.02	0.50	3.77
41	(Heart failure, Cerebral infarction)	=>	(Atherosclerotic heart disease)	0.02	0.58	4.77
